# A High-Performing Plasma Metabolite Panel for Early-Stage Lung Cancer Detection

**DOI:** 10.3390/cancers12030622

**Published:** 2020-03-07

**Authors:** Lun Zhang, Jiamin Zheng, Rashid Ahmed, Guoyu Huang, Jennifer Reid, Rupasri Mandal, Andrew Maksymuik, Daniel S. Sitar, Paramjit S. Tappia, Bram Ramjiawan, Philippe Joubert, Alessandro Russo, Christian D. Rolfo, David S. Wishart

**Affiliations:** 1 Department of Biological Sciences, University of Alberta, Edmonton, AB T6G 2E8, Canada; lun2@ualberta.ca (L.Z.); jiamin3@ualberta.ca (J.Z.); jreid3@ualberta.ca (J.R.); rmandal@ualberta.ca (R.M.); 2 BioMark Diagnostics Inc., Richmond, BC V6X 2W8, Canada; Rashid.biomark@gmail.com (R.A.); Gina.biomark@gmail.com (G.H.); 3 Cancer Care Manitoba, Winnipeg, MB R3E 0V9, Canada; amaksymiuk@cancercare.mb.ca; 4 Department of Internal Medicine, Rady Faculty of Health Sciences, University of Manitoba, Winnipeg, MB R3A 1R9, Canada; Daniel.Sitar@umanitoba.ca; 5 Department of Pharmacology & Therapeutics, Rady Faculty of Health Sciences, University of Manitoba, Winnipeg, MB R3E 0T5, Canada; 6 Asper Clinical Research Institute & Office of Clinical Research, St. Boniface Hospital, Winnipeg, MB R2H 2A6, Canada; ptappia@sbrc.ca (P.S.T.); BRamjiawan@sbrc.ca (B.R.); 7 Department of Pathology, University of Laval, Quebec, QC G1V 4G5, Canada; philippe.joubert.1@ulaval.ca; 8 Medical Oncology Unit A.O. Papardo & Department of Human Pathology, University of Messina, 98158 Messina, Italy; ale.russo1986@gmail.com; 9 Thoracic Medical Oncology Program Marlene and Stewart Greenebaum Comprehensive Cancer Center, University of Maryland, Baltimore, MD 21201, USA; christian.rolfo@umm.edu

**Keywords:** lung cancer, early detection, cancer staging, metabolomics, LC-MS

## Abstract

The objective of this research is to use metabolomic techniques to discover and validate plasma metabolite biomarkers for the diagnosis of early-stage non-small cell lung cancer (NSCLC). The study included plasma samples from 156 patients with biopsy-confirmed NSCLC along with age and gender-matched plasma samples from 60 healthy controls. A fully quantitative targeted mass spectrometry (MS) analysis (targeting 138 metabolites) was performed on all samples. The sample set was split into a discovery set and validation set. Metabolite concentration data, clinical data, and smoking history were used to determine optimal sets of biomarkers and optimal regression models for identifying different stages of NSCLC using the discovery sets. The same biomarkers and regression models were used and assessed on the validation models. Univariate and multivariate statistical analysis identified β-hydroxybutyric acid, LysoPC 20:3, PC ae C40:6, citric acid, and fumaric acid as being significantly different between healthy controls and stage I/II NSCLC. Robust predictive models with areas under the curve (AUC) > 0.9 were developed and validated using these metabolites and other, easily measured clinical data for detecting different stages of NSCLC. This study successfully identified and validated a simple, high-performing, metabolite-based test for detecting early stage (I/II) NSCLC patients in plasma. While promising, further validation on larger and more diverse cohorts is still required.

## 1. Introduction

Lung cancer is the leading cause of cancer-related deaths worldwide, with an estimated 1.69 million individuals dying each year [1]. Despite significant advances in treatment, survival rates for lung cancer have largely remained unchanged for the past 40 years [2,3,4]. However, when lung cancer is detected and resected in its earliest stages (stage I), the 10-year survival rate is increased to >80% [5]. Therefore, sensitive and accurate strategies for the early detection of lung cancer are essential if we wish to improve lung cancer survival statistics. Unfortunately, current methods for the detection or screening of lung cancer are not ideal. While low dose computed tomography (LDCT) screening has been shown to reduce lung cancer mortality [6,7], broad clinical implementation is hampered by several technical and socioeconomical challenges. Therefore, the development of a low-cost, minimally invasive assay for early stage lung cancer detection would significantly improve the current situation.

Over the past 20 years a number of blood-based lung cancer assays that detect protein [8,9], microRNA [10], circulating DNA [11,12,13], and methylated DNA [14] biomarkers have been developed. Unfortunately, most are specific to late-stage lung cancer [9,10,13]. More recently researchers have turned to the analysis of metabolite biomarkers for lung cancer detection [15]. Indeed, it is now well established that cancer cells produce distinct chemical signatures that can be seen in tissues and biofluids [16]. As a result, a number of metabolite biomarkers that are specific to lung cancer have been discovered in a variety of biofluids, including serum/plasma, bronchial fluid, or sputum [17,18,19]. These biomarkers have frequently been discovered via metabolomics. Metabolomics combines advanced analytical chemistry techniques with cheminformatics to characterize thousands of metabolites found in tissues and biofluids. While these metabolomic studies of lung cancer have demonstrated promising areas under the curve (AUC) (ranging from 0.78 and 0.95) [20], most were aimed at detecting late-stage lung cancer. Furthermore, many of these studies were conducted with small samples sizes, without absolute metabolite quantification and without validation in larger cohorts. To the best of our knowledge, there are only a handful of metabolomic studies that used quantitative metabolomics to detect early-stage lung cancer that included both biomarker discovery and subsequent validation [21,22,23]. Unfortunately, these studies did not succeed in finding very high performing biomarkers (with AUCs < 0.8).

Here we describe a quantitative metabolomic study that has succeeded in discovering and validating a set of high performing (AUC > 0.9) plasma metabolite biomarkers for detecting early stage non-small cell lung cancer (NSCLC). NSCLC represents the major subtype of lung cancer (about 80–85%) [1]. In conducting this study, we performed absolute quantitative LC-MS metabolomics analysis of plasma samples acquired from 156 patients with biopsy-proven and biopsy-graded NSCLC and 60 healthy controls from the same age and gender-matched cohort. The cancer cohort included 70 stage I, 60 stage II, and 26 stage IIIB/IV samples (Table 1). Both univariate and multivariate statistical analysis were performed to discover differences in the metabolite profiles between a subset of NSCLC patients and healthy controls. Robust predictive models that used these plasma biomarkers were then built using logistic regression. The metabolite biomarkers and logistic regression models were then confirmed and validated in a separate hold-out (i.e., validation) set. These models, which included metabolites only or metabolites plus clinical data, consistently achieved AUCs > 0.9 for detecting different stages of NSCLC.

## 2. Materials and Methods

### 2.1. Regulatory and Institutional Review Board Approvals

Ethics approval was obtained from the University of Manitoba Health Research Ethics Board (Ethics File #: H2012:334) prior to study implementation. We also received research ethics approval from the University of Alberta (Study ID: Pro00093715) to perform the metabolomic studies in Edmonton.

### 2.2. Study Population

Archived plasma samples were obtained from the IUCPQ (Institut Universitaire de Cardiologie et de Pneumologie de Quebec) Tissue Bank, which is the site of the Respiratory Health Network Tissue Bank of the Fonds de la Recherché du Quebec-Sante in Quebec, Canada. Dates of sample collection range from 2005 to 2017. Frozen (−80 °C) aliquots of 200–400 μL of plasma were assembled and shipped to The Metabolomic Innovation Centre (TMIC) at the University of Alberta, Canada for quantitative metabolomic analysis. The plasma samples were collected from 156 patients with biopsy-proven and biopsy-graded NSCLC and 60 healthy controls with comparable age and gender profiles. Healthy controls consisted of both smokers and non-smokers. The cancer samples had detailed data on cancer stage, lung cancer histology, age, weight, height, body mass index, smoking status (never/former/current), smoking history (cig/day and period of smoking in years), sex, survival history, medical condition history, personal history of cancer, lung disease status, treatment, tumor size (in mm), tumor grading, details of positive nodules, as well as data collected on each cancer patient’s transthoracic needle biopsy, transbronchial biopsy, endobronchial biopsy, bronchoalveolar lavage, bronchial brushing, bronchial aspiration, endobronchial ultrasound, transesophageal echocardiography, bone scintigraphy, abdominal ultrasound, abdominal CT scan, thoracic CT scan, cerebral CT scan, thoracic X-ray, mediastinoscopy, thoracic MRI, cerebral MRI, and PET scan. Healthy controls had data on age, weight, height, body mass index, smoking status (never/former/current), smoking history (cig/day and period of smoking in years), and medical condition history. Patients (and controls) with a history of any liver or kidney disease, and any previous treatment with anti-neoplastic drugs were excluded from this cohort.

### 2.3. Chemicals, Reagents, and Materials for Metabolomic Assays

Optima™ LC/MS grade formic acid and HPLC grade water were purchased from Fisher Scientific (Ottawa, ON, Canada). Sixty-eight pure reference standard compounds were purchased from Sigma-Aldrich (Oakville, ON, Canada). Optima™ LC/MS grade Ammonium acetate, phenylisothiocyanate (PITC), 3-nitrophenylhydrazine (3-NPH), 1-ethyl-3-(3-dimethylaminopropyl) carbodiimide (EDC) and butylated hydroxytoluene (BHT), HPLC grade pyridine, HPLC grade methanol, HPLC grade ethanol, and HPLC grade acetonitrile (ACN) were also purchased from Sigma-Aldrich (Oakville, ON, Canada). Forty-four ^2^H-, ^13^C-, and ^15^N-labelled compounds, which were used as internal quantification standards for amino acids, biogenic amines, carnitines and derivatives, phosphatidylcholines and their derivatives were purchased from Cambridge Isotope Laboratories, Inc. (Tewksbury, MA, USA). 3-(3-hydroxyphenyl)-3-hydroxypropionic acid (HPHPA) and ^13^C-labelled HPHPA were synthesized in-house as described previously [24]. All other standards including lactic acid, beta-hydroxybutyric acid, alpha-ketoglutaric acid, citric acid, butyric acid, isobutyric acid, propionic acid, p-hydroxyhippuric acid, succinic acid, fumaric acid, pyruvic acid, hippuric acid, methylmalonic acid, homovanillic acid, indole-3-acetic acid, uric acid, and their isotope-labelled standards were all purchased from Sigma-Aldrich (Oakville, ON, Canada). Multiscreen “solvinert” filter plates (hydrophobic, PTFE, 0.45 μm, clear, non-sterile) and Nunc^®^ 96 DeepWell™ plates were purchased from Sigma-Aldrich (Oakville, ON, Canada).

### 2.4. Stock Solutions, Internal Standard (ISTD) Mixture, and Calibration Curve Standards for Metabolomic Assays

All solid chemicals were carefully weighed on a CPA225D semi-micro electronic balance (Sartorius, NY, USA) with a precision of 0.0001 g. Stock solutions of each compound were prepared by dissolving the accurately weighed solids in double-distilled water. Calibration curve standards were obtained by mixing and diluting the corresponding stock solutions with double-distilled water. For amino acids, biogenic amines, carbohydrates, carnitines and derivatives, phosphatidylcholines and their derivatives, stock solutions of isotope-labelled compounds were also prepared in the same way. A working internal standard (ISTD) solution mixture in water was also made by mixing all the prepared isotope-labeled stock solutions together. For organic acids, stock solutions of isotope-labelled compounds were prepared by dissolving the accurately weighed solids in 75% aqueous methanol. A working internal standard (ISTD) solution mixture in 75% aqueous methanol was made by mixing and diluting all the isotope-labelled stock solutions. All standard solutions were aliquoted and stored at −80 °C until further use.

### 2.5. Sample Preparation and Liquid Chromatography/Direct Injection Mass Spectrometry for Metabolomic Assays

A targeted, quantitative mass spectrometry (MS)-based metabolomics approach was used to analyze the plasma samples using a combination of direct injection (DI) MS and reverse-phase high performance liquid chromatography (HPLC) tandem mass spectrometry (MS/MS). This 96-well plate, semi-automated assay, in combination with an ABI 4000 Q-Trap (Applied Biosystems/MDS Sciex) mass spectrometer, can be used for the targeted identification and quantification of up to 138 different endogenous metabolites including amino acids, organic acids, biogenic amines, acylcarnitines, glycerophospholipids, sphingolipids, and sugars. The method combines the derivatization and extraction of the 138 analytes, and the selective mass-spectrometric detection using multiple reaction monitoring (MRM) pairs. Isotope-labeled internal standards and other internal standards are integrated into special filter inserts placed inside a 96-well plate for precise metabolite quantification. The assay uses an upper 96 deep-well plate with a 96-well filter plate attached below using sealing tape. The first 14 wells in the upper plate are used for quality control and calibration. The first well serves as a double blank, three wells contain blank samples, seven wells contain reference compound standards, and three wells contain quality control samples.

Briefly, plasma samples were thawed on ice (in the dark) and were vortexed and centrifuged at 18,000 rcf (relative centrifugal force or × *g*). Then, 10 µL of each sample was loaded onto the center of the filter insert on the upper 96-well kit plate and dried in a stream of nitrogen. Subsequently, PITC was added to each well (in the plate for amine derivatization. After incubation, the filter inserts were dried using an evaporator. Extraction of the metabolites was then achieved by adding 300 µL of methanol containing 5 mM ammonium acetate. The extracts were obtained by centrifugation (at 50 rcf for 5 min) of the double plate system. This allowed the contents of the upper 96-well plate to flow into the lower 96-deep well plate. For analysis of biogenic amines and amino acids, extracts were then diluted by water. For analysis of sugars, carnitines, and lipids, extracts were diluted with methanol. Mass spectrometric analysis of the diluted extracts was performed on an HPLC (Agilent 1100 HPLC, Agilent Technologies, Santa Clara, CA, USA) equipped Qtrap^®^ 4000 tandem mass spectrometry instrument (Applied Biosystems/MDS Analytical Technologies, Foster City, CA, USA).

For the analysis of organic acids, 50 μL of the plasma samples were mixed thoroughly with the ISTD mixture solution and ice-cold methanol and then left in a −20 °C freezer overnight for protein precipitation. After removing the samples from the freezer, all the tubes were centrifuged at 18,000 rpm for 20 min (to spin down the protein precipitate). The supernatant was then transferred to each well of the 96-well plate system, followed by the addition of 25 μL each of the following three reagents: 3-NPH (250 mM in methanol), EDC (150 mM in methanol), and pyridine for a 2 h derivatization reaction. After the derivatization reaction was complete, water and a BHT solution (2 mg/mL in methanol) were added to dilute and stabilize the final solution. Finally, 10 μL was injected into an HPLC-equipped Qtrap^®^ 4000 mass spectrometer for LC-MS/MS analysis.

### 2.6. Statistical Analysis

Recommended statistical procedures for standard quantitative metabolomic analysis were followed [25]. In quantitative metabolomic studies, missing values normally indicate that the metabolite fell below the assay’s limit of detection (LOD). Therefore, metabolites with more than 50% of missing values (in all groups) were removed from further analysis. For metabolites with the fraction of missing values below 50%, values were imputed by using half of the minimum concentration value for that metabolite. Median normalization, log transformation, and auto-scaling (mean-centered and divided by the standard deviation of each variable) were applied for data scaling and normalization. Univariate analysis of the continuous data and the categorical data were performed by a Mann–Whitney rank sum test and a Fisher’s exact test, respectively. Principal component analysis (PCA) and partial least squares discriminant analysis (PLS-DA) were performed by using MetaboAnalyst [8]. A 1000-fold permutation test was performed to minimize the possibility that the observed separation of the PLS-DA was due to chance.

Logistic regression with a Lasso feature selection algorithm was used to develop predictive models of NSCLC staging using both metabolite and clinical variables. For these regression studies, two thirds of the samples (40 controls, and between 40 and 94 cancer samples, depending on staging) were randomly chosen to serve as the discovery sets. Then, 10-fold cross validation was performed on all discovery/training set models. Once the optimal regression models for each cancer stage predictor had been identified the remaining one third of the samples (20 controls and between 20 and 62 cancer samples, serving as a holdout set) were used to validate each of the corresponding regression models. The area under the receiver-operator characteristic curves (AUC), sensitivities/specificities at selected cut-off points and the 95% confidence intervals were calculated for all of the discovery and the validation sets and all of the models using MetaboAnalyst [8]. Cut-off points were selected by calculating the Youden Index (J = max {Sensitivity + Specificity − 1}).

## 3. Results

### 3.1. Statistical Data Processing

A total of 138 different metabolites were tested by our quantitative LC-MS method. Due to their low abundance in serum and plasma, 35 metabolites were found to have a high (>50%) fraction of missing values. Therefore, these metabolites were removed from the further analysis, as is standardly done in most quantitative metabolomic studies [25]. No statistically significant difference (as measured by a Mann–Whitney rank sum test) between cancer cases and healthy controls was observed among these 35 low-abundance metabolites. Sample numbers in each group are summarized in Table 1.

### 3.2. Statistical Analysis on Clinical Variables

Comparisons between the cancer patients and healthy controls regarding age, gender, height, weight, and smoking history (Yes = former + current, No = never) were conducted using standard Student’s *t*-tests or Fisher’s exact test to confirm their demographic comparability. The only significantly different variable was smoking history (*p*-value = 2.673 × 10^−13^). The effect on lung cancer incidence based on multiple clinical variables, including age, gender, height, weight, and smoking history (Yes = former + current, No = never) was further evaluated by logistic regression. The results are summarized in Table S1. As might be expected, only smoking history was identified as the clinical variable significantly related to lung cancer incidence (*p*-value = 1.13 × 10^−11^). Although the correlation between smoking history and lung cancer has been heavily studied and widely accepted, our model suggested it would be a good strategy to integrate smoking history (including duration and amount of smoking) into any diagnostic model for identifying early lung cancer.

### 3.3. Statistical Analysis: Normal vs. NSCLC at All Stages

By applying a Mann–Whitney rank sum test to our metabolomics data set, large differences between the metabolic profiles of healthy controls and lung cancer patients (all stages) were revealed. Table S2 lists the 39 metabolites with significant FDR adjusted *p*-values (q < 0.05) identified via the Mann–Whitney rank sum test. In our study, phosphatidylcholines such as PC ae C40:6, PC aa C38:0, and PC aa C40:2 were among the most downregulated metabolites in the plasma of NSCLC patients, while lysophosphatidylcholines (LysoPCs) such as LysoPC 20:3 and LysoPC 18:2 were significantly upregulated in cancer patients. Other significantly altered metabolites included β-hydroxybutyric acid (increased in NSCLC), carnitines (C0 and C2, both increased), tryptophan (decreased), methionine sulfoxide (decreased), and members of the TCA cycle such as citrate (decreased) and fumaric acid (increased).

Multivariate analysis was also conducted to further reveal metabolite differences between healthy controls and NSCLC patients at all stages. Using PLS-DA we found a clear separation between NSCLC patients and healthy controls (Figure S1a). Permutation testing demonstrated that the observed separation was not by chance (*p* < 0.001). LysoPC 20:3, carnitine, β-hydroxybutyric acid, and PC ae C40:6 were found to have the highest overall coefficient score that drove the separation (Figure S1b).

### 3.4. Multivariate Analysis: Stage I vs. Normal

**Figure 1:**
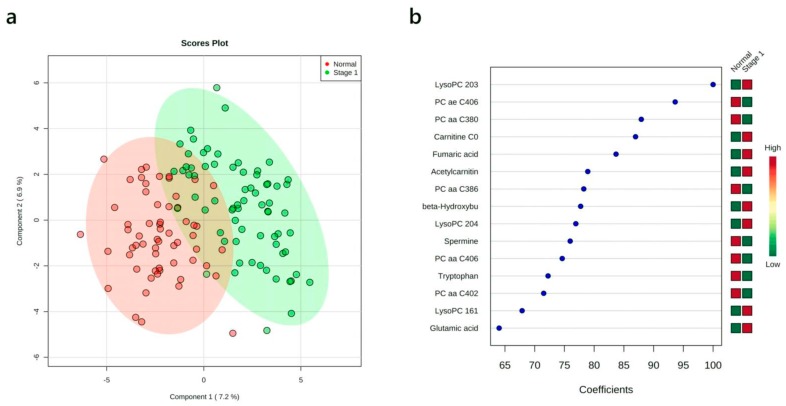
Partial least squares discriminant analysis (PLS-DA) results showing the comparison between plasma metabolite data acquired for healthy controls vs. stage I non-small cell lung cancer (NSCLC) patients. (**a**) 2-D PLS-DA scores plots; (**b**) variable importance in projection plot. The most discriminating metabolites are shown in descending order of their coefficient scores. The color boxes indicate whether metabolite concentration is increased (red) or decreased (green) in controls vs. cases.

Biomarkers that can effectively diagnose lung cancer patients’ early stages of the disease are obviously more valuable than biomarkers for later stages of the disease. Therefore, we carried out a series of statistical analyses to identify plasma metabolites that could distinguish NSCLC patients at stage I vs. healthy controls. As shown in Figure 1a the PLS-DA analysis shows a clearly detectable separation between the stage I NSCLC group and healthy controls (Figure 1a). Permutation testing revealed that the observed separation between the cases and controls was not due to chance (*p*-value < 0.001). Figure 1b displays the results of the overall coefficient scores from the PLS-DA. Based on this analysis, LysoPC 20:3, PC ae C40:6, PC aa C38:0, carnitine, and fumaric acid appeared to be the most important plasma metabolites for distinguishing stage I NSCLC patients from healthy controls.

**Figure 2:**
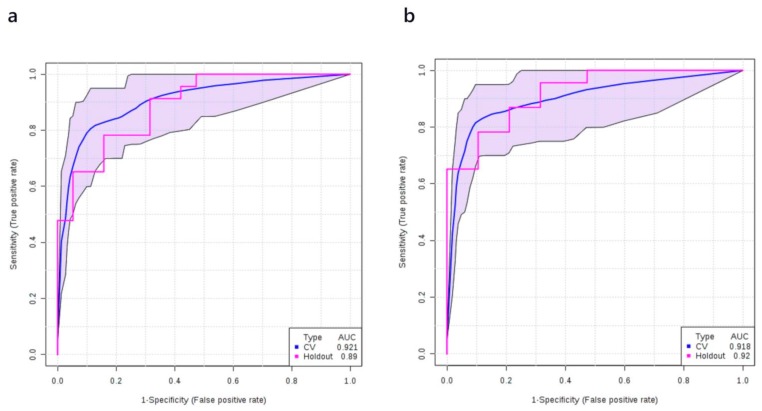
Receiver-operating characteristic (ROC) curve generated by the logistic regression models for diagnosing stage I NSCLC patients. (**a**) ROC curve of the metabolite-only model; (**b**) ROC curve of the metabolites + smoking model. ROC curves and their 95% CI on the discovery set are shown in blue. ROC curves obtained from the validation set are colored in red.

Logistic regression along with random forest based exploratory receiver-operating characteristic (ROC) analysis was performed using MetaboAnalyst to identify the best metabolite combination to distinguish stage I NSCLC from healthy controls. In this analysis, balanced sub-sampling-based Monte Carlo cross validation (MCCV) was used to generate the ROC curves. Using a discovery cohort of plasma samples from 40 healthy controls and 47 stage I NSCLC patients we found that the AUC of different ROC models with different numbers of metabolite features ranged from 0.824 to 0.922 (Figure S2a). Figure S2b shows the most frequently selected metabolites with LysoPC 20:3, PC ae C40:6, PC aa C38:0, LysoPC 20:4, fumaric acid, carnitine, and β-hydroxybutyric acid being identified as the top-listed metabolites. A logistic regression model was then built to predict the probability of having stage I NSCLC (P) with the following equation: log(P/(1 − P)) = 0.258 − 1.341 × PC ae C40:6 + 1.747 × LysoPC 20:3 + 0.913 × β-hydroxybutyric acid + 0.939 × Fumaric acid, where the numeric value of each named metabolite in the equation is the concentration after median normalization, log transformation, and auto-scaling. Specifically, the values for each named metabolite are scaled as follows: PC ae C40:6 = log_10_([PC ae C40:6]/4.85)/0.18; LysoPC 20:3 = (log_10_([LysoPC 20:3]/4.34) − 0.05)/0.18; β-hydroxybutyric acid = (log_10_([β-hydroxybutyric acid]/47.1) − 0.1)/0.45; Fumaric acid = (log_10_([Fumaric acid]/0.91) − 0.02)/0.2. Here and in all other models below, [PC ae C40:6], [LysoPC 20:3], [β-hydroxybutyric acid], and [Fumaric acid] represent the measured plasma concentrations (in µM) of PC ae C40:6, LysoPC 20:3, β-hydroxybutyric acid, and fumaric acid, respectively. The ROC curve with 95% confidence interval (CI) is shown in Figure 2a. The AUC and the 10-fold cross-validation AUC of the ROC curve was 0.939 (95% CI, 0.924–0.955) and 0.923 (95% CI, 0.866–0.980), respectively. The performance of the metabolite-only model was further checked on the validation set (which consisted of 20 healthy controls and 23 stage I cancer patients) and a slightly lower AUC was obtained (0.890). The ROC curve obtained from the validation set is shown in Figure 2a as well. Other details of the model are listed in Table 2.

When the smoking history of patients was added, our logistic model for the discovery cohort was modified to logit(P) = log(P/(1 − P)) = 0.311 + 0.641 × Amount of smoking − 1.372 × PC ae C40:6 + 1.623 × LysoPC 20:3 + 0.882 × β-hydroxybutyric acid + 0.65 × Fumaric acid, where P is the probability of stage I NSCLC. As before, the numeric value of each named metabolite in the above equation is the concentration after median normalization, log transformation, and auto-scaling. Specifically, the values for each named metabolite are scaled as follows: PC ae C40:6 = log_10_([PC ae C40:6]/4.85)/0.18; LysoPC 20:3 = (log_10_([LysoPC 20:3]/4.34) − 0.05)/0.18; β-hydroxybutyric acid = (log_10_([β-hydroxybutyric acid]/47.1) − 0.1)/0.45; Fumaric acid = (log_10_([Fumaric acid]/0.91) − 0.02)/0.2; Amount of smoking = (log_10_([Amount of smoking]/4106) + 1.52)/2.29. Here and in all other models below, [PC ae C40:6], [LysoPC 20:3], [β-hydroxybutyric acid], and [Fumaric acid] represent the measured plasma concentrations (in µM) of PC ae C40:6, LysoPC 20:3, β-hydroxybutyric acid and fumaric acid, respectively. Additionally, [Amount of smoking] was calculated by multiplying the period of smoking (in days) by the daily amount of smoking (pack/day). The ROC curve of the corresponding model is shown in Figure 2b. The AUC for the metabolite + smoking model was 0.942 (95% CI, 0.926–0.957) and after 10-fold cross-validation it was 0.922 (95% CI, 0.864–0.979). This was similar to the metabolite-only model. When the same metabolite + smoking history model was tested on the validation set, the AUC of the validation cohort was essentially the same (0.920, Figure 2b) as the metabolite-only model. Interestingly, the Youden index for the cut-off point (0.74) was modestly increased when smoking history was taken into consideration (Table 3).

### 3.5. Multivariate Analysis: Stage II vs. Normal

**Figure 3:**
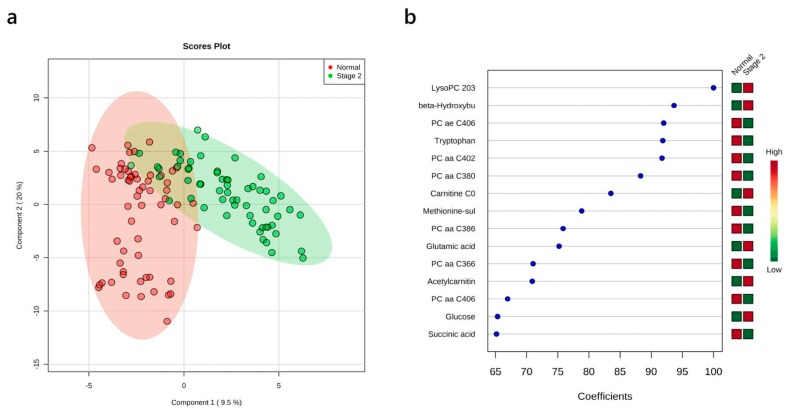
PLS-DA results showing the comparison between plasma metabolite data acquired for healthy controls vs. stage II NSCLC patients. (**a**) 2-D PLS-DA scores plots; (**b**) variable importance in projection plot. The most discriminating metabolites are shown in descending order of their coefficient scores. The color boxes indicate whether metabolite concentration is increased (red) or decreased (green) in controls vs. cases.

A similar series of analyses was carried out for lung cancer patients at stage II. The corresponding PLS-DA plot along with the VIP plot are shown in Figure 3a,b. Permutation testing revealed that the observed separation of the cases from the normal group was not due to chance (*p*-value < 0.001). Comparing with NSCLC patients at stage I, fumaric acid was no longer identified as one of the most important features in the PLS-DA VIP plot, while β-hydroxybutyric acid was identified as one of the metabolites with the highest coefficient score.

**Figure 4:**
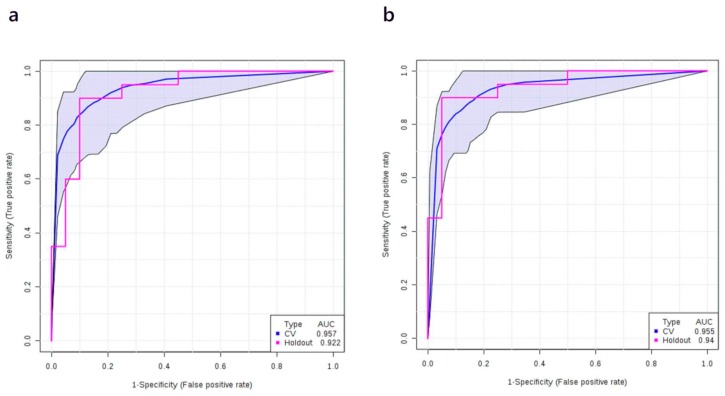
ROC curve generated by the logistic regression models for stage II NSCLC patients. (**a**) ROC curve of the metabolites-only model; (**b**) ROC curve of the metabolites + smoking history model. ROC curves and their 95% CI on the discovery set are shown in blue. ROC curves obtained from the validation set are colored in red.

Using a discovery cohort of plasma samples consisting of 40 healthy controls and 40 stage II NSCLC patients we found that the AUC of different metabolite-only regression models with different numbers of metabolite features ranged from 0.894 to 0.946 (Figure S3a). Figure S3b shows the most frequently selected metabolites. LysoPC 20:3, tryptophan, β-hydroxybutyric acid, PC ae C40:6, glutamic acid, and carnitine were identified as the most differentiating metabolites. A logistic regression model was then built to predict the probability of having stage II NSCLC (P) with the following equation: logit(P) = log(P/(1 − P)) = 0.346 + 2.565 × β-hydroxybutyric acid − 2.219 × Citric acid + 2.904 × Carnitine − 1.599 × PC ae C40:6, where the numeric value of each named metabolite in the equation is the concentration after median normalization, log transformation, and auto-scaling. Specifically, β-hydroxybutyric acid = (log_10_([β-hydroxybutyric acid]/50.75) − 0.14)/0.5; Citric acid = (log_10_([Citric acid]/89.65) + 0.02)/0.16; Carnitine = (log_10_([Carnitine]/31.89) + 0.06)/0.21; PC ae C40:6 = (log_10_([PC ae C40:6]/4.82 + 0.01)/0.19. Here and in all other models below, [PC ae C40:6], [β-hydroxybutyric acid], [Citric acid], and [Carnitine] represent the plasma concentrations (in µM) of citric acid and carnitine, respectively. The ROC curve with its 95% CI is shown in Figure 4a. The AUC and the 10-fold cross-validation AUC of the ROC curve is 0.980 (95% CI, 0.973–0.987) and 0.952 (95% CI, 0.909–0.995), respectively. The performance of the metabolite-only model was further checked on the holdout validation set (which consisted of 20 healthy controls and 20 stage II cancer patients) and a slightly lower AUC was obtained (0.922). The ROC curve obtained from the validation set is shown in Figure 4a as well. Other details of the model are listed in Table 4.

When the smoking history of patients was added, the logistic model for the discovery cohort was modified to logit(P) = log(P/(1 − P)) = 0.098 + 1.489 × Amount of smoking + 2.911 × β-hydroxybutyric acid − 1.627 × Citric acid + 2.605 × Carnitine − 0.702 × PC ae C40:6, where P is the probability of stage II NSCLC and the numeric value of each named metabolite in the equation is the concentration after median normalization, log transformation, and auto-scaling. Specifically, β-hydroxybutyric acid = (log_10_([β-hydroxybutyric acid]/50.75) − 0.14)/0.5; Citric acid = (log_10_([Citric acid]/89.65) + 0.02)/0.16; Carnitine = (log_10_([Carnitine]/31.89) + 0.06)/0.21; PC ae C40:6 = (log_10_([PC ae C40:6]/4.82 + 0.01)/0.19; Amount of smoking = (log_10_([Amount of smoking]/4106) + 1.31)/2.3. As before, values in square brackets represent measured (unscaled) concentrations of the compounds. The ROC curve of the corresponding model is shown in Figure 4b. The AUC of the ROC curve for the metabolite + smoking model was 0.985 (95% CI, 0.979–0.991) and after 10-fold cross-validation it was 0.948 (95% CI, 0.900–0.996). When the same metabolite + smoking history model was tested on the validation set, AUC of the validation set was also close to the training set (0.940, Figure 4b). Similar to the model for stage I NSCLC, the Youden index for the cut-off point (0.25) and the overall model performance on the validation set was improved when smoking history was taken into consideration (Table 5).

### 3.6. Multivariate Analysis: Stage I+II vs. Healthy Controls

**Figure 5:**
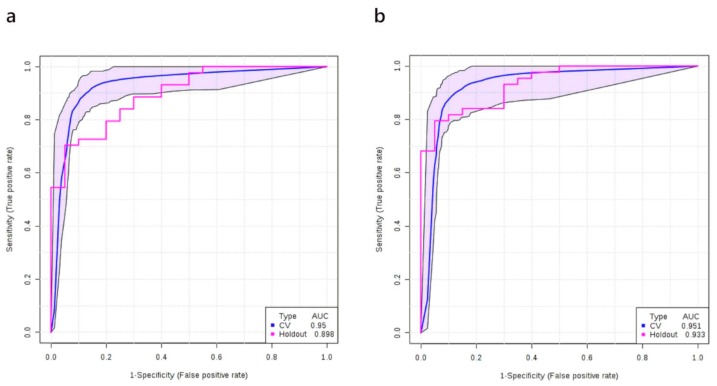
ROC curve generated by the logistic regression models for NSCLC patients at early stages (stage I + II). (**a**) ROC curve of the metabolites-only model; (**b**) ROC curve of the metabolites + smoking history model. ROC curves and their 95% CI on the discovery set are shown in blue. ROC curves obtained from the validation set are colored in red.

We applied the same methods described above to obtain a predictive model for diagnosing stage I+II NSCLC patients together (defined as early stage NSCLC). Using a discovery cohort of plasma samples from 40 healthy controls and 87 early stage NSCLC patients, we built a logistic regression model to predict the probability of having early stage NSCLC (P) with the following equation: logit(P) = log(P/(1 − P)) = 2.346 − 1.528 × PC ae C40:6 + 1.429 × β-hydroxybutyric acid − 2.481 × Citric acid + 1.03 × LysoPC 20:3 + 1.773 × Fumaric acid, where the numeric value of each named metabolite in the equation is the concentration after median normalization, log transformation and auto-scaling. Specifically, PC ae C40:6 = (log_10_([PC ae C40:6]/4.27) + 0.02)/0.18; β-hydroxybutyric acid = (log_10_([β-hydroxybutyric acid]/58.1) − 0.11)/0.48; LysoPC 20:3 = (log_10_([LysoPC 20:3]/4.27) + 0.04)/0.16; Citric acid = (log_10_([Citric acid]/86.9) + 0.01)/0.14; Fumaric acid = (log_10_([Fumaric acid]/0.93) − 0.01)/0.2. As before, values in square brackets represent measured (unscaled) concentrations of the compounds. The ROC curve with its 95% CI is shown in Figure 5a. The AUC and the 10-fold cross-validation AUC of the ROC curve was 0.974 (95% CI, 0.965–0.982) and 0.959 (95% CI, 0.923–0.995), respectively. The performance of the metabolite-only model was further checked on the validation set (which consisted of 20 healthy controls and 43 early-stage patients) and a slightly lower AUC was obtained (0.898). The ROC curve obtained from the validation set and other details of the model are shown in Figure 5a and Table 6, respectively.

When the smoking history of patients was added, the logistic model for the discovery cohort was modified to logit(P) = log(P/(1 − P)) = 2.427 + 1.425 × Amount of smoking − 1.414 × PC ae C40:6 + 1.414 × β-hydroxybutyric acid − 2.193 × Citric acid + 1.738 × LysoPC 20:3 + 1.44 × Fumaric acid, where P is the probability of stage II NSCLC and the numeric value of each named metabolite in the equation is the concentration after median normalization, log transformation, and auto-scaling. Specifically, PC ae C40:6 = (log_10_([PC ae C40:6]/4.27) + 0.02)/0.18; β-hydroxybutyric acid = (log_10_([β-hydroxybutyric acid]/58.1) − 0.11)/0.48; LysoPC 20:3 = (log_10_([LysoPC 20:3]/4.27) + 0.04)/0.16; Citric acid = (log_10_([Citric acid]/86.9) + 0.01)/0.14; Fumaric acid = (log_10_([Fumaric acid]/0.93) − 0.01)/0.2; Amount of smoking = (log_10_([Amount of smoking]/6570) + 1.23)/2.1. As before, values in square brackets represent measured (unscaled) concentrations of the compounds. The ROC curve of the corresponding model is shown in Figure 5b. The AUC of the ROC curve for the metabolite + smoking model was 0.982 (95% CI, 0.975–0.990) and after 10-fold cross-validation it was 0.948 (95% CI, 0.930–1.000). When the same metabolite + smoking history model was tested on the validation set, the AUC of the validation set was reasonably close to the training set (0.933, Figure 5b). Again, when smoking history was added into the model, both the sensitivities/specificities of the cut-off point (0.66) and overall model performance were improved (Table 7).

### 3.7. Multivariate Analysis: Stages IIIB+IV vs. Normal

Metabolite analysis of the plasma of patients at advanced stages of NSCLC were much more distinct from healthy controls, compared with earlier NSCLC stages. Both PCA and PLS-DA responded with clear separation (Figure S4a,b). The VIP data from the PLS-DA analysis showed that ketone body dysregulation appeared to be one of the most characteristic features of stage IIIB+IV NSCLC patients (Figure S4c). Elevated levels of cadaverine, a product of lysine decarboxylation, was also identified as one of the most important features in discriminating stages IIIB+IV NSCLC. In contrast, upregulation of LysoPC 20:3, which was a feature of stage I/II NSCLC did not stand out as an important feature in stage III/IV NSCLC. As the identification of markers for late stage lung cancer was not a major focus of this work (and because of the relatively small sample size), we did not attempt to develop a logistic regression model to predict stage IIIB/IV NSCLC.

## 4. Discussion

The purpose of this study was to discover and validate a combination of plasma metabolite (and clinical) biomarkers for the early detection of non-small cell lung cancer (NSCLC). In particular, plasma metabolite changes in NSCLC patients (at various stages) versus healthy (age and gender-matched) controls were studied via quantitative MS-based metabolomic techniques. Separate discovery cohorts (with 10-fold cross validation) and validation cohorts were used to prevent overtraining and any unintended bias in the results. Three different metabolite-only and three different metabolite + smoking status models were developed and independently validated to detect stage I, stage II, and stage I/II NSCLC. Most of these models achieved AUCs > 0.9. Figure S5 shows a Venn diagram representing discovered plasma metabolite biomarkers for specific stages.

Over the past decade, a large number of metabolomic studies have been published aimed at identifying robust biomarkers for lung cancer diagnosis using plasma, serum, or urine. Regardless of the lung cancer staging, most of these studies were performed on relatively small sample sizes (*n* < 50 in each group), most were not validated with an independent holdout group and many were based on qualitative (i.e., non-quantitative) metabolomic methods [20,26,27,28,29,30]. For a metabolite assay to be clinically useful, the metabolite measurements must be fully quantitative. After an extensive literature review, we found remarkably few metabolomic studies that specifically looked at detecting early stage lung cancer, that used reasonably large cohorts, and which employed fully quantitative metabolomic techniques. In particular just three studies met some or all of these criteria. The study by Maeda et al., which was performed in 2010, described the development of an early stage lung cancer detection test based on precisely determined concentrations of 21 plasma amino acids [21]. The test was developed using a cohort of 4340 healthy controls and 186 patients with stage I/II (162 in stage I and 24 in stage II) lung cancer and the final model used only six amino acids. The authors reported AUCs of 0.817 and 0.801 (on their validation sets) for diagnosing stage I and stage II lung cancer, respectively. We attempted to repeat this result using our assay and our cohort as we measured all the plasma amino acids described by Maeda et al. [21]. Interestingly, using the same panel of six amino acids, we were able to generate logistic regression models with AUCs of 0.774 and 0.878 for stage I and stage II NSCLC, respectively. While we do not know the exact equations used by Maeda et al. [21] in their diagnostic model, we believe our results largely validate their findings and confirm the importance of plasma amino acids in diagnosing early stage lung cancer. However, the use of additional metabolite classes (LysoPCs, organic acids) and other clinical data certainly can improve the diagnostic performance, as our classifiers for both stage I and II NSCLC had AUCs of 0.921 and 0.957, respectively.

A recent lipidomics study conducted by Yu et al. discovered that a combination of four plasma lipids could be used to detect early-stage NSCLC [22]. This semi-quantitative test was developed using a cohort of 80 healthy controls and 105 patients with stage I/II NSCLC. Their initial model had an AUC of 0.823. When the assay was validated on an independent cohort the AUC was 0.808. These results are comparable to those reported by Maeda et al. and they certainly suggest that lipids have a useful role to play in diagnosing early stage lung cancer. Unfortunately, the lipids measured in the Yu et al. study were not measurable in our study, so we could not independently confirm their findings. A more recent study conducted by Ros-Mazurczyk et al. described a LysoPC-based serum assay for the diagnosis of early stage NSCLC, which had an AUC = 0.88 [23]. This semi-quantitative test was developed using a cohort of 300 healthy controls and 94 patients in stage I/II NSCLC. However, the assay was not validated on a separate cohort. It also needs to be pointed out that the high AUC claimed in the Ros-Mazurczyk study was based on the semi-quantitation of seven unidentified LysoPCs. When the assay was limited to the four identifiable LysoPCs the AUC dropped to 0.80. We also attempted to repeat the Ros-Mazurczyk result using our assay and our cohort as we quantitatively measured all four of the identified LysoPCs described in their paper. Our AUC was just 0.675 for stage I/II NSCLC. The reduced performance we obtained may be related to with intrinsic differences in LysoPC levels for plasma vs. serum [31,32]. Furthermore, specificity (76%) and PPV (55%) of the Ros-Mazurczyk model are surprisingly low, especially when compared to our results which typically have specificities > 90%, and PPVs of ~90%.

As noted above, the diagnostic accuracy of all previously reported metabolomics assays for detecting early-stage lung cancer is relatively modest (with AUCs ≈ 0.8). This may be due to the limited number and type of metabolites (amino acids only, lipids only, LysoPCs only, etc.) that were quantitatively or semi-quantitatively measured. The plasma biomarker panels we discovered for stage I, stage II, and stage I/II lung cancer cover a more diverse range of metabolites (lipids, LysoPCs, organic acids, amino acids) and this may be why the performance is consistently better (AUCs > 0.9).

We have earlier determined a liquid biopsy panel consisting of 14 metabolites, six of which are in the polyamine pathway, that was able to correctly diagnose lung cancer at later stages with an area under the curve of 0.97 (95% CI: 0.875–1.0) [33]. The present study was subsequently undertaken with an increase in the range of metabolites to allow for the detection and determination of lung cancer at early stages. While the performance of our metabolite-only or metabolite + smoking models for diagnosing early stage NSCLC is quite impressive, these models still need to be further validated on larger, more diverse cohorts. In particular, a much larger age and gender-matched healthy control group along with a more ethnically diverse population would be helpful to determine if these metabolite signatures are truly robust and if they provide sufficient sensitivity/specificity for lung cancer screening purposes. Currently, we are planning to extend and validate these models on a larger cohort containing 1200–1500 patients.

In addition for further validation on a larger cohort, the inclusion of individuals with other pulmonary diseases (pneumonia, tuberculosis, chronic obstructive pulmonary disease (COPD), asthma) in a separate control group would also help determine whether these metabolite markers are specific to lung cancer alone or whether they are also markers for general lung distress. To explore the issue of lung distress vs. lung cancer we conducted a literature review of serum/plasma metabolomic studies that have looked at these other lung conditions and found that the markers we identified do not overlap with the markers identified for these conditions [34,35,36,37,38]. This suggests that the metabolite markers we found are likely specific to lung cancer, but this supposition clearly needs further experimental validation. The inclusion of other clinical variables beyond smoking status/history could also improve the performance of our models. For instance, data on ethnicity, coughing frequency, incidence of respiratory tract infections, occupational exposure to dust/powders, location relative to known areas with high radon, etc., could be used to assist in lung cancer diagnosis.

While the development and validation of a simple and reliable plasma metabolite assay was the primary purpose of this study, it is also of interest to try to understand why some of the metabolites we found were so differentially expressed in NSCLC. By comparing the predictive models acquired from stage I NSCLC patients to models for other stages, LysoPC 20:3 was identified as a key metabolic biomarker for stage I NSCLC. As yet, no published study has previously reported on the lung cancer-related biological functions of LysoPC 20:3. In our univariate study, plasma concentrations of this lysophosphatidylcholine acquired from NSCLC patients at all stages showed a clear elevation with significant FDR adjusted *p*-values (*p* < 0.01). This observation linking elevated levels of LysoPC 20:3 to stage I/II NSCLC was further confirmed by our multivariate statistical studies and subsequence predictive models. It has been observed and reported that plasma concentrations of total LysoPCs are often inversely correlated with the risk of various types of cancer in both the mouse [39] and human [40,41] models. Our univariate analysis of total LysoPCs also revealed that in stages IIIB and IV NSCLC, plasma levels of most of the measured LysoPCs were downregulated (data not shown). In particular, LysoPC 18:0 and LysoPC 17:0 showed a significant decrease (FDR adjusted *p* = 0.0103 and 0.0028, respectively), which is consistent with previous reports [39,41]. Lower levels of most LysoPCs may be related to the higher consumption rate of LysoPCs and LysoPC-bound fatty acids in tumor cells [42], and the increased rate of extracellular LysoPC cleavage [39]. Given the opposing trend of LysoPC 20:3 (increased) compared with other LysoPCs (decreased), we propose that LysoPC 20:3 may have a unique role in the development/progression of lung cancer. In particular, LysoPCs have been implicated in phagocyte recruitment and opsonization of apoptotic cells [43]. An increased plasma level of LysoPC 20:3 may be related to an alternated immune response at early stages of NSCLC.

Another member of phosphatidylcholine family, PC ae C40:6, also appears to play a role in both stage I and stage II NSCLC. In this study, concentrations of polyunsaturated PCs with 38–40 carbons were found to be significantly decreased in the plasma of NSCLC patients. The inclusion of PC ae C40:6, in our predictive models for early stage NSCLC contributed significantly to the high sensitivity/specificity of our models. While the precise lipid species associated with PC ae C40:6 could not be determined, it is possible that PC ae C40:6 could be the source for LysoPC 20:3, thereby explaining the significant reduction of PC ae C40:6 compared to the significant increase in LysoPC 20:3. Altered plasma PC levels in early-stage NSCLC patients have been previously reported [44]. It has been noted previously that decreased lipid membrane unsaturation levels can protect tumor cells from free radicals or chemotherapeutics and promote invasion and infiltration [45]. Decreased polyunsaturated PC levels have been previously reported in six different types of cancer tissues including lung cancer [46]. Clearly more detailed lipidomic studies need to be conducted to investigate the biological significance of these PC alterations.

Elevated plasma levels of β-hydroxybutyric acid, one of the most abundant plasma ketone bodies, was found for NSCLC at all stages. Consistent with our results, several other recently published studies have detected the same trend in plasma β-hydroxybutyric acid from lung cancer patients [47,48,49]. The biological functions of β-hydroxybutyric acid are quite diverse and include energy metabolism, epigenetic regulation, and oxidative stress response [50]. Different studies in animal models and humans on the biological effects of β-hydroxybutyric acid in cancer have often led to diverse conclusions. Therefore, the relationship between β-hydroxybutyric acid and cancer, especially lung cancer, has yet to be clarified. The elevated plasma β-hydroxybutyric acid in stage IIIB and IV patients may be due to cancer-related malnutrition. However, changes in β-hydroxybutyric acid at earlier lung cancer stages is more difficult to explain. It has been recently reported that extra β-hydroxybutyric acid can be produced by nearby or adjacent fibroblasts to feed tumor cells [51]. Therefore, the elevated plasma β-hydroxybutyric acid in lung cancer patients may be a resulting overproduction of β-hydroxybutyric acid by tumors or adjacent tumor tissue. More studies are needed to bring to light the precise reasons why plasma β-hydroxybutyric acid is upregulated in lung cancer patients.

Increased plasma fumaric acid levels were identified as a powerful biomarker to discriminate stage I NSCLC patients from healthy controls, which is another novel finding. Another component of the TCA cycle, citric acid, also contributed to the predictive models for stage II and advanced stage NSCLC. Both of these TCA organic acids were found to be altered in plasma across all NSCLC stages but with opposite trends (fumarate increased while citrate decreased). Fumaric acid has been linked to the inhibition HIF-1α degradation in tumor cells to overcome hypoxia in multiple cancer types [52,53,54]. In this regard fumaric acid can be considered as an oncometabolite [55]. On the other hand, citric acid can be used by tumor cells as a source of acetyl-CoA and oxaloacetic acid for lipid synthesis and neoglucogenesis [56]. Fumaric acid and citric acid have been shown to be rapidly taken up by lung cancer cells, and their accumulation in lung cancer tissue has also been shown by a number of different studies [57,58]. Decreased plasma citrate levels in lung cancer has been described previously [59], which helps confirm the importance of this compound in lung cancer diagnosis. However, the rationale for the opposing trends of these two metabolites in the plasma of lung cancer patients still needs further clarification.

In our study, plasma carnitine levels were significantly increased—across all NSCLC stages. Increased production of carnitine has also been identified as a significant feature of plasma, tumor tissue, and other types of biofluids acquired from NSCLC patients [60] as well as patients with other types of cancer, such as bladder [61], breast [62], and colorectal cancer [63]. Carnitine is rapidly consumed by tumor cells for acetyl-CoA synthesis and acetyl-CoA can be used for lipid synthesis via glutamine metabolism [56,64,65]. Endogenous carnitines in mammals are mainly synthesized in liver and kidney via trimethyllysine, a product of lysine methylation [64]. In our study, another product of lysine metabolism, cadaverine, was also found to be significantly increased in the plasma of stage IIIB and IV NSCLC patients. These abnormal trends in carnitine and cadaverine levels suggest that dysregulation of lysine metabolism is a common feature of NSCLC, especially in more advanced stages.

## 5. Conclusions

In summary, we h developed several high-performing logistic regression models for the diagnosis of early stage NSCLC using plasma metabolites that consistently have AUCs > 0.9. Both metabolite-only and metabolite + smoking history models were developed on an initial discovery set and then fully validated on a separate holdout set. In all cases the Youden index for the cut-off points was improved by incorporating smoking duration. A key advantage of developing a blood-based metabolomic test is that it can be easily converted into a low-cost, high-throughput assay that can be run at almost any clinical laboratory equipped with a standard triple-quadrupole mass spectrometer. We estimate that a modified assay that is specific to the metabolites identified here could be run at a rate of 4–5 min per sample using as little as 10 µL of plasma. These promising results suggest that a minimally invasive, high performance, high-throughput, low cost lung cancer screening assay might be developed that could be used to select patients for further follow-up and confirmation using LDCT or other lung imaging modalities. Future validation studies involving larger cohorts, additional clinical parameters, and the inclusion of patients with other lung diseases as negative controls are being undertaken.

## Figures and Tables

**Table 1:** Summary of grouping of samples.

**Discovery Set**											
**Group**	**Number of Samples**	**Age**		**Histology**		**Gender**		**Smoking Status**			
		**Range**	**Median**	**Adenocarcinoma**	**Squamous Cell Carcinoma**	**Male**	**Female**	**Never**	**Former**	**Current**	Median Pack * Years (Former + Current)
Stage I NSCLC	47	49–79	66	32	15	18	29	10	26	11	36
Stage II NSCLC	40	49–79	61.5	29	11	11	29	3	34	3	34
Stage IIIB/IV NSCLC	26	42–79	63	20	6	14	12	0	18	8	43
Healthy control	40	49–77	62.5	NA	NA	18	22	25	15	0	11
Total	153	42–79	64	81	32	61	92	57	131	28	33
**Validation Set**											
**Group**	**Number of Samples**	**Age**		**Histology**		**Gender**		**Smoking Status**			
		**Range**	**Median**	**Adenocarcinoma**	**Squamous Cell Carcinoma**	**Male**	**Female**	**Never**	**Former**	**Current**	Median Pack * Years (Former + Current)
Stage I NSCLC	23	49–78	65	18	5	8	15	4	14	5	35
Stage II NSCLC	20	51–78	64	11	9	9	11	2	16	2	38
Healthy control	20	49–77	62.5	NA	NA	8	12	13	7	0	5
Total	63	49–78	65	29	14	25	38	57	131	28	27

* 1 pack = 20 cigarettes.

**Table 2:** Logistic regression based optimal model for stage I NSCLC detection: metabolites only.

**Logistic Model with Selected Compounds:**							
log(P/(1 − P)) = 0.258 − 1.341 × PC ae C40:6 + 1.747 × LysoPC 20:3 + 0.913 × β-hydroxybutyric acid + 0.939 × Fumaric acid. The optimal cut-off point for the above equation is 0.69.							
**Logistic Regression Model—Summary of Each Feature:**							
	**Estimate**	**Std. Error**		**z Value**	**Pr(>|z|)**		**Odds**
**(Intercept)**	0.258	0.352		0.733	0.463		-
**LysoPC 20:3**	1.747	0.518		3.37	0.001		5.73
**β-Hydroxybutyric acid**	0.913	0.404		2.263	0.024		2.49
**Fumaric acid**	0.939	0.446		2.106	0.035		2.56
**PC ae C40:6**	−1.341	0.465		−2.884	0.001		0.26
**Performance of Logistic Regression Model:**							
	**AUC**		**Sensitivity**			**Specificity**	
**Training/discovery**	0.939 (0.924–0.955)		0.827 (0.791–0.863)			0.957 (0.936–0.977)	
**10-fold cross-validation**	0.923 (0.866–0.980)		0.830 (0.830–0.937)			0.927 (0.847–1.000)	

**Table 3:** Logistic regression based optimal model for stage I NSCLC detection: metabolites plus smoking history.

**Logistic Model with Selected Compounds:**							
logit(P) = log(P/(1 − P)) = 0.311 + 0.641 × Amount of smoking − 1.372 × PC ae C40:6 + 1.623 × LysoPC 20:3 + 0.882 × β-hydroxybutyric acid + 0.65 × Fumaric acid. The optimal cut-off point for the above equation is 0.74.							
**Logistic Regression Model—Summary of Each Feature:**							
	**Estimate**	**Std. Error**		**z Value**	**Pr(>|z|)**		**Odds**
**(Intercept)**	0.311	0.369		0.843	0.399		-
**Amount of smoking**	0.641	0.382		1.676	0.094		1.9
**PC ae C40:6**	−1.372	0.475		−2.886	0.004		0.25
**LysoPC 20:3**	1.623	0.495		3.281	0.001		5.07
**β-Hydroxybutyric acid**	0.882	0.419		2.105	0.035		2.42
**Fumaric acid**	0.65	0.474		1.373	0.17		1.92
**Performance of Logistic Regression Model:**							
	**AUC**		**Sensitivity**			**Specificity**	
**Training/discovery**	0.942 (0.926–0.957)		0.844 (0.809–0.879)			0.951 (0.929–0.973)	
**10-fold cross-validation**	0.922 (0.864–0.979)		0.851 (0.851–0.953)			0.951 (0.882–1.000)	

**Table 4:** Logistic regression based optimal model for stage II NSCLC detection: metabolites only.

**Logistic Model with Selected Compounds:**							
logit(P) = log(P/(1 − P)) = 0.346 + 2.565 × β-hydroxybutyric acid − 2.219 × Citric acid + 2.904 × Carnitine − 1.599 × PC ae C40:6. The optimal cut-off point for the above equation is 0.34.							
**Logistic Regression Model—Summary of Each Feature:**							
	**Estimate**	**Std. Error**		**z Value**	**Pr(>|z|)**		**Odds**
**(Intercept)**	0.346	0.516		0.671	0.502		-
**β-Hydroxybutyric acid**	2.565	0.861		2.981	0.003		13.93
**Citric acid**	−2.219	0.804		−2.758	0.006		0.11
**Carnitine**	2.904	0.976		2.975	0.003		18.24
**PC ae C40:6**	−1.599	0.765		−2.091	0.037		0.2
**Performance of Logistic Regression Model:**							
	**AUC**		**Sensitivity**			**Specificity**	
**Training/discovery**	0.980 (0.973–0.987)		0.958 (0.938–0.979)			0.881 (0.854–0.909)	
**10-fold cross-validation**	0.952 (0.909–0.995)		0.875 (0.875–0.977)			0.875 (0.773–0.977)	

**Table 5:** Logistic regression based optimal model for stage II NSCLC detection: metabolites plus smoking history.

**Logistic Model with Selected Compounds:**							
logit(P) = log(P/(1 − P)) = 0.098 + 1.489 × Amount of smoking + 2.911 × β-hydroxybutyric acid − 1.627 × Citric acid + 2.605 × Carnitine − 0.702 × PC ae C40:6. The optimal cut-off point for the above equation is 0.25.							
**Logistic Regression Model—Summary of Each Feature:**							
	**Estimate**	**Std. Error**		**z Value**	**Pr(>|z|)**		**Odds**
**(Intercept)**	−0.098	0.612		0.159	0.873		-
**Amount of smoking**	1.489	0.915		1.627	0.104		4.43
**β-Hydroxybutyric acid**	2.911	1.132		2.572	0.01		18.37
**Citric acid**	−1.627	0.864		−1.883	0.06		0.2
**Carnitine**	2.605	0.936		2.784	0.005		13.53
**PC ae C40:6**	−0.702	0.862		−0.814	0.416		0.5
**Performance of Logistic Regression Model:**							
	**AUC**		**Sensitivity**			**Specificity**	
**Training/discovery**	0.985 (0.979–0.991)		0.972 (0.955–0.989)			0.875 (0.841–0.909)	
**10-fold cross-validation**	0.948 (0.900–0.996)		0.925 (0.925–1.000)			0.850 (0.739–0.961)	

**Table 6:** Logistic regression based optimal model for stages I + II NSCLC detection: metabolites only.

**Logistic Model with Selected Compounds:**							
logit(P) = log(P/(1 − P)) = 2.346 − 1.528 × PC ae C40:6 + 1.429 × β-hydroxybutyric acid − 2.481 × Citric acid + 1.03 × LysoPC 20:3 + 1.773 × Fumaric acid. The optimal cut-off point for the above equation is 0.62.							
**Logistic Regression Model—Summary of Each Feature:**							
	**Estimate**	**Std. Error**		**z Value**	**Pr(>|z|)**		**Odds**
**(Intercept)**	2.346	0.588		3.991	<0.001		-
**PC ae C40:6**	−1.528	0.61		−2.507	0.012		0.22
**β-Hydroxybutyric acid**	1.429	0.505		2.832	0.005		4.18
**Citric acid**	−2.481	0.642		−3.863	<0.001		0.08
**LysoPC 20:3**	1.03	0.508		2.028	0.043		2.8
**Fumaric acid**	1.773	0.569		3.117	0.002		5.89
**Performance of Logistic Regression Model:**							
	**AUC**		**Sensitivity**			**Specificity**	
**Training/discovery**	0.974 (0.965–0.982)		0.937 (0.920–0.954)			0.922 (0.895–0.950)	
**10-fold cross-validation**	0.959 (0.923–0.995)		0.919 (0.919–0.976)			0.900 (0.807–0.993)	

**Table 7:** Logistic regression based optimal model for stages I + II NSCLC detection: metabolites plus smoking history.

**Logistic Model with Selected Compounds:**							
logit(P) = log(P/(1 − P)) = 2.427 + 1.425 × Amount of smoking − 1.414 × PC ae C40:6 + 1.414 × β-hydroxybutyric acid − 2.193 × Citric acid + 1.738 × LysoPC 20:3 + 1.44 × Fumaric acid. The optimal cut-off point for the above equation is 0.66.							
**Logistic Regression Model—Summary of Each Feature:**							
	**Estimate**	**Std. Error**		**z Value**	**Pr(>|z|)**		**Odds**
**(Intercept)**	2.427	0.638		3.803	<0.001		-
**Amount of smoking**	1.425	0.507		2.813	0.005		4.16
**PC ae C40:6**	−1.048	0.64		−1.637	0.102		0.35
**β-Hydroxybutyric acid**	1.414	0.594		2.379	0.017		4.11
**Citric acid**	−2.193	0.719		−3.051	0.002		0.11
**LysoPC 20:3**	1.738	0.739		2.351	0.019		5.68
**Fumaric acid**	1.44	0.612		2.352	0.019		4.22
**Performance of Logistic Regression Model:**							
	**AUC**		**Sensitivity**			**Specificity**	
**Training/discovery**	0.982 (0.975–0.990)		0.960 (0.946–0.974)			0.944 (0.921–0.968)	
**10-fold cross-validation**	0.965 (0.930–1.000)		0.930 (0.930–0.984)			0.925 (0.843–1.000)	

## Supplementary Materials

The following are available online at https://www.mdpi.com/2072-6694/12/3/622/s1, Figure S1: PLS-DA results of healthy controls vs. all stages NSCLC, Figure S2: ROC curve of the random forest exploration models for stage I NSCLC patients, Figure S3: ROC curve of the random forest exploration models for stage II NSCLC patients, Figure S4: PCA and PLS-DA results of healthy controls vs. Stages IIIB+IV NSCLC, Figure S5: Venn Diagram representing discovered plasma metabolite markers for different early stages of NSCLC, Table S1: Logistic regression based correlation study: NSCLC vs. clinical variants, Table S2: Metabolites with significant different between normal cases and NSCLC patients using univariate statistical analysis (Mann Whitney Rank Sum test).
