# Supplementary material for: A High-Performing Plasma Metabolite Panel for Early-Stage Lung Cancer Detection
Source: Cancers (Basel). 2020 Mar 7;12(3):622. doi: 10.3390/cancers12030622 (PMC7139410; doi:10.3390/cancers12030622)
Supplement: Supplementary file 1 [file cancers-12-00622-s001.pdf]

cancers

Supplementary Materials:

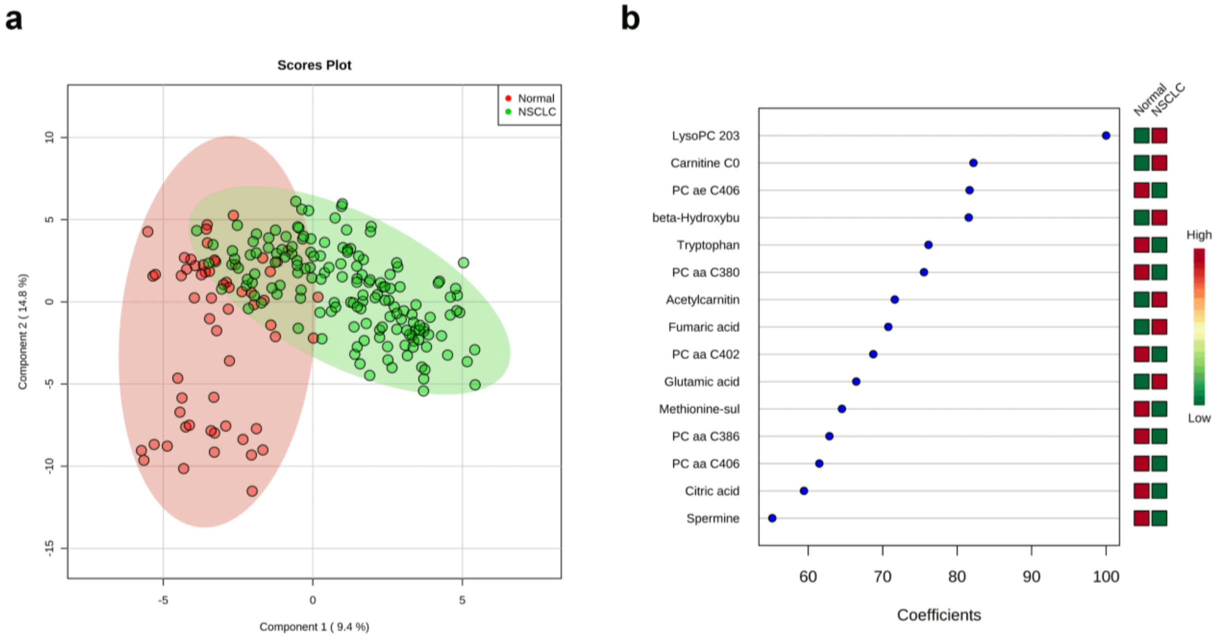

Figure S1. PLS-DA results of healthy controls vs. all stages NSCLC. (**a**) 2-D PLS-DA scores plots; (**b**) Variable importance in projection plot. The most discriminating metabolites are shown in descending order of coefficient scores. The color boxes indicate whether metabolite concentration is increased (red) or decreased (green) in controls vs. cases.

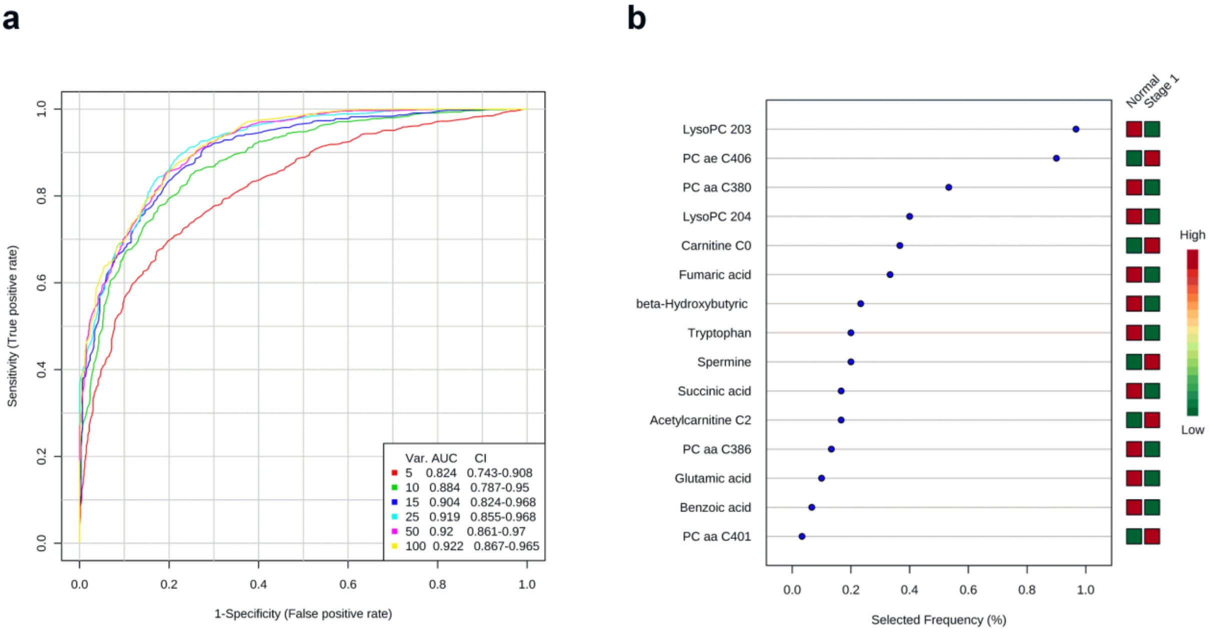

Figure S2. ROC curve of the random forest exploration models for stage I NSCLC patients. (**a**) ROC curves with different numbers of metabolite features. Number of metabolite features in each model are indicated as Var. in the left-bottom box; (**b**) The most frequently selected metabolites (Number of features = 5). The color boxes indicate whether metabolite concentration is increased (red) or decreased (green) in controls vs. cases.

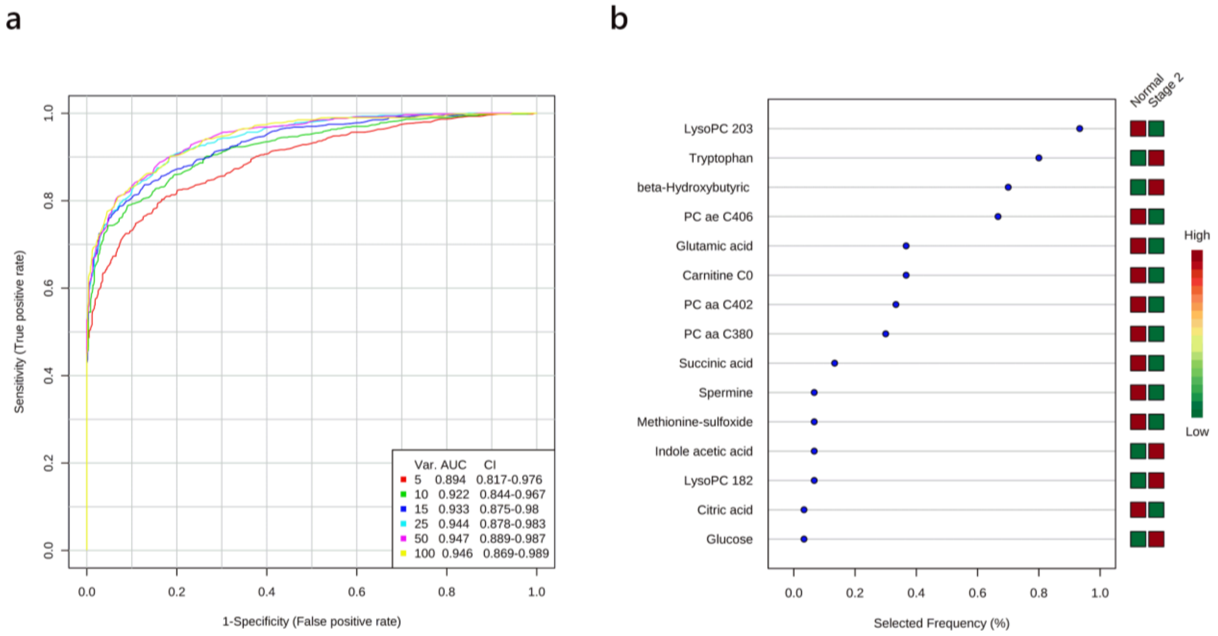

Figure S3. ROC curve of the random forest exploration models for stage II NSCLC patients. (**a**) ROC curves with different numbers of metabolite features. Number of metabolite features in each model are indicated as Var. in the left-bottom box; (**b**) The most frequently selected metabolites (Number of features = 5). The color boxes indicate whether metabolite concentration is increased (red) or decreased (green) in controls vs. cases.

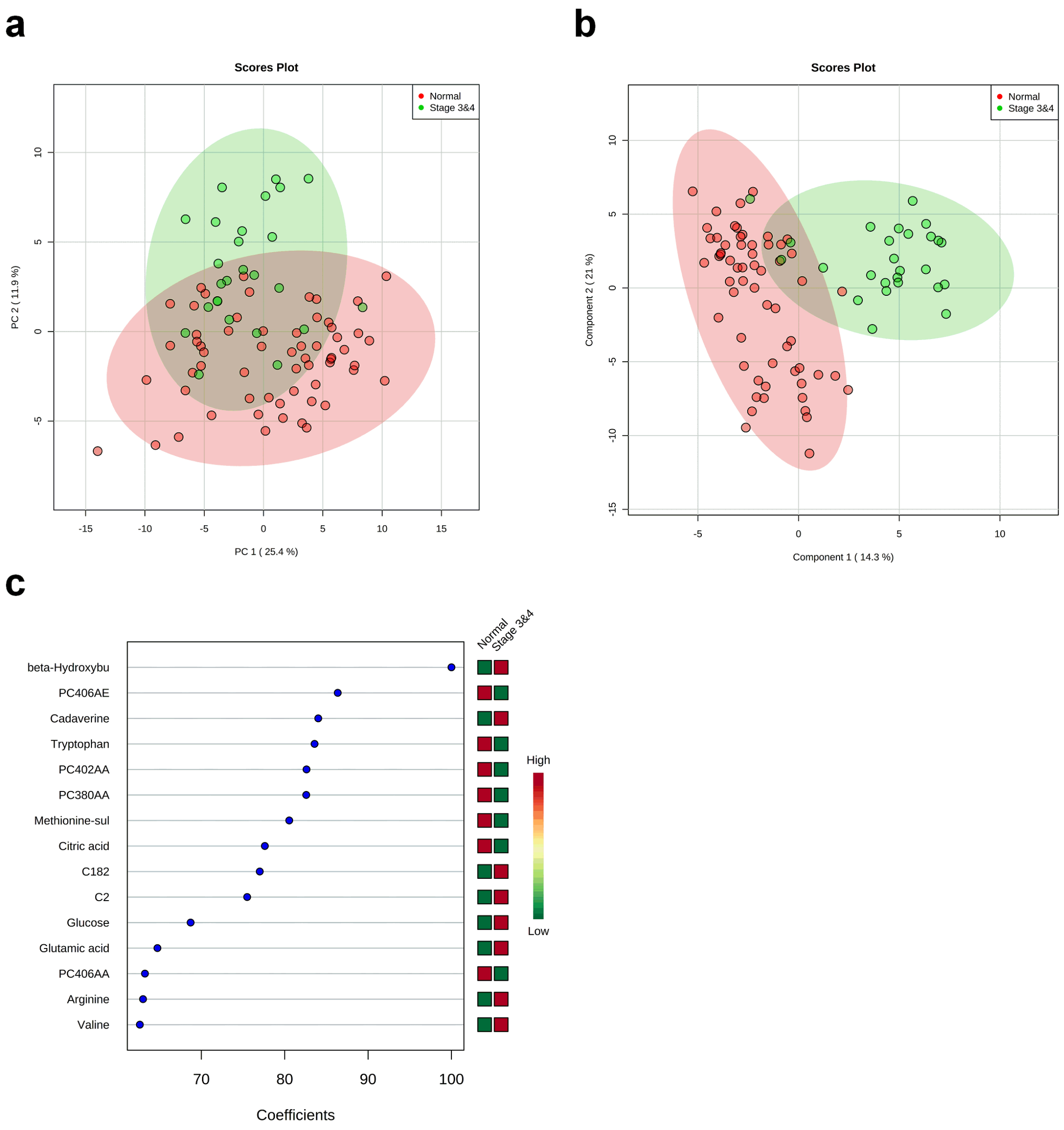

Figure S4. PCA and PLS-DA results of healthy controls vs. Stages IIIB+IV NSCLC. (**a**) 2-D PCA scores plots; (**b**) 2-D PLS-DA scores plots; (**c**) Variable importance in projection plot. The most discriminating metabolites are shown in descending order of coefficient scores. The color boxes indicate whether metabolite concentration is increased (red) or decreased (green) in controls vs. cases.

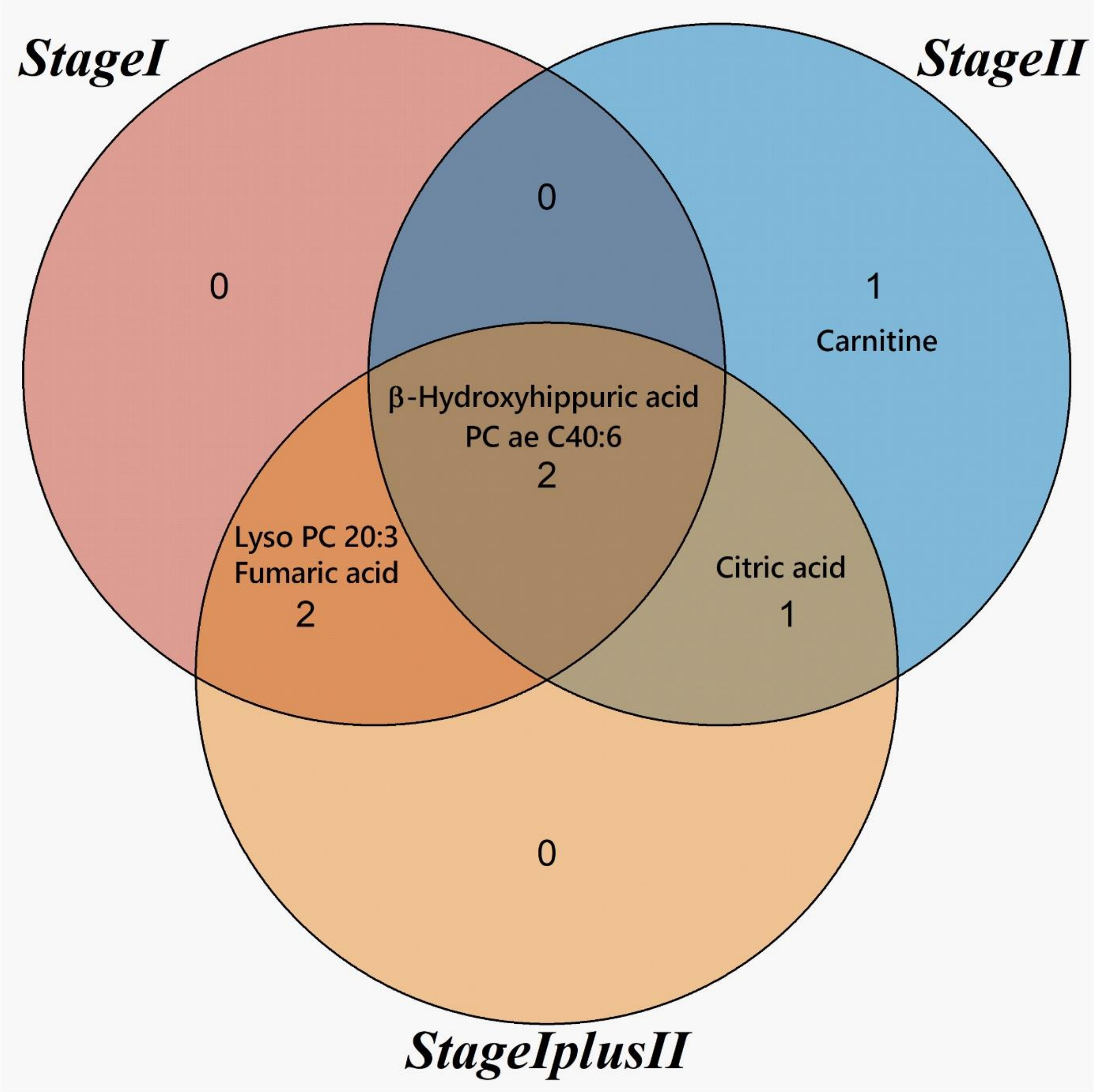

Figure S5. Venn Diagram representing discovered plasma metabolite markers for different early stages of NSCLC.

Table S1. Logistic regression based correlation study: NSCLC vs. clinical variants.

	Estimate	std.error	z value	*p*-value
**(Intercept)**	5.9317	5.6113	1.0571	0.2905
**Age**	0.0170	0.0232	0.7306	0.4650
**Gender**	−0.2930	0.5870	−0.4991	0.6177
**Height**	−4.6740	3.3846	−1.3810	0.1673
**Weight**	−0.0014	0.0138	−0.1041	0.9171
**Smoking (Y/N)**	2.8079	0.4136	6.7883	1.13×10^{-11}

Table S2. Metabolites with significant different between normal cases and NSCLC patients using univariate statistical analysis (Mann Whitney Rank Sum test).

Name of Metabolites	Fold Chang (Normal/Case)	*p*-value	FDR
**PC ae C40:6**	1.35	2.09×10^{-12}	2.32×10^{-10}
**LysoPC 20:3**	0.73	7.73×10^{-12}	4.29×10^{-10}
**β-hydroxybutyric acid**	0.25	1.20×10^{-11}	4.44×10^{-10}
**PC aa C38:0**	1.32	3.56×10^{-11}	9.88×10^{-10}
**Carnitine**	0.71	1.00×10^{-9}	2.22×10^{-08}
**Tryptophan**	1.46	3.57×10^{-9}	6.00×10^{-8}
**PC aa C40:2**	0.69	4.02×10^{-9}	6.00×10^{-8}
**Acetylcarnitine**	1.33	4.32×10^{-9}	6.00×10^{-8}
**Methionin × 10–sulfoxide**	1.52	4.14×10^{-8}	5.11×10^{-7}
**PC aa C38:6**	1.30	5.37×10^{-8}	5.96×10^{-7}
**Glutamic acid**	0.66	2.21×10^{-7}	2.23×10^{-6}
**PC aa C40:6**	1.28	9.52×10^{-7}	8.80×10^{-6}
**PC aa C36:6**	1.31	3.99×10^{-6}	3.40×10^{-5}
**Succinic acid**	1.16	7.35×10^{-6}	5.83×10^{-5}
**Citric acid**	1.29	1.15×10^{-5}	8.54×10^{-5}
**Fumaric acid**	0.71	2.36×10^{-5}	1.63×10^{-4}
**Spermine**	1.18	3.43×10^{-5}	2.24×10^{-4}
**Glucose**	0.79	4.80×10^{-5}	2.96×10^{-4}
**Valine**	0.78	1.08×10^{-4}	6.34×10^{-4}
**Indole acetic acid**	1.17	1.84×10^{-4}	1.02×10^{-3}
**Tyrosine**	1.25	4.93×10^{-4}	2.61×10^{-3}
**C18:2**	0.74	9.85×10^{-4}	4.91×10^{-3}
**SM (OH) C14:1**	1.14	1.02×10^{-3}	4.91×10^{-3}
**C6:1**	0.71	1.80×10^{-3}	8.10×10^{-3}
**Pyruvic acid**	0.81	1.83×10^{-3}	8.10×10^{-3}
**LysoPC 18:2**	1.22	1.97×10^{-3}	8.28×10^{-3}
**C18**	1.15	2.01×10^{-3}	8.28×10^{-3}
**Ornithine**	0.82	2.45×10^{-3}	9.70×10^{-3}
**LysoPC 20:4**	0.82	5.58×10^{-3}	2.13×10^{-2}
**Creatine**	1.13	5.95×10^{-3}	2.16×10^{-2}
**Alanine**	1.17	6.02×10^{-3}	2.16×10^{-2}
**LysoPC 16:1**	0.83	6.36×10^{-3}	2.21×10^{-2}
**Arginine**	0.80	8.21×10^{-3}	2.76×10^{-2}
**Betaine**	1.27	8.94×10^{-3}	2.92×10^{-2}
**SM (OH) C24:1**	1.12	9.74×10^{-3}	3.09×10^{-2}
**Cadaverine**	0.80	1.02×10^{-2}	3.13×10^{-2}
**Trimethylamine N-oxide**	1.79	1.19×10^{-2}	3.56×10^{-2}
**C10**	1.53	1.45×10^{-2}	4.24×10^{-2}
**Diacetylspermine**	0.90	1.55×10^{-2}	4.42×10^{-2}
